# Risk factors for elder abuse severity: findings from the Canadian longitudinal study on aging

**DOI:** 10.1093/geroni/igaf101

**Published:** 2025-09-23

**Authors:** David Burnes, Clémentine Rotsaert, Mark S Lachs, Karl A Pillemer

**Affiliations:** Factor-Inwentash Faculty of Social Work, University of Toronto, Toronto, Ontario, Canada; Faculty of Arts and Science, University of Toronto, Toronto, Ontario, Canada; Geriatrics and Palliative Medicine, Weill Cornell Medicine, New York, New York, United States; College of Human Ecology, Cornell University, Ithaca, New York, United States

**Keywords:** CLSA, Mistreatment, Life course perspective, Ecological

## Abstract

**Background and Objectives:**

Elder abuse (EA) conceptualizations are evolving from conventional binary understandings toward a severity lens that more accurately captures the spectrum of victim experiences and complexity of EA intervention. Although momentum behind a focus on severity has grown, our understanding of EA severity risk factors is methodologically limited by studies using clinical convenience samples and/or cross-sectional designs. Informed by the Contextual Theory of Elder Abuse, this article sought to advance the state of science surrounding EA severity risk factors using data from a longitudinal, population-based design and examining factors from several levels of ecological influence.

**Research Design and Methods:**

Using the Canadian Longitudinal Study on Aging, this study analyzed a sample of EA victims (*n *= 2,364) reporting past-year emotional/psychological, physical, and/or financial abuse, who completed baseline and follow-up waves of data collection. EA victimization was assessed using validated tools and behaviorally defined items describing specific mistreatment behaviors. Calculation of EA severity for each subtype combined dimensions of behavioral *multiplicity* (number of mistreatment behaviors) and *frequency*. Independent change variables were used to confirm the direction of change underlying potential risk factors prior to EA victimization. Multinomial logistic regression was used to identify factors associated with increased levels of EA severity.

**Results:**

Across subtypes, the most consistent risk factors for heightened EA severity were perpetrator cohabitation and the older adult’s experience of child maltreatment. Other risk factors were identified across physical, psycho-emotional, social, and sociocultural domains. Risk profiles varied across mistreatment subtypes.

**Discussion and Implications:**

This study represents the most methodologically rigorous examination of EA severity risk conducted to date. Findings will enhance our capacity to identify EA victims in particularly harmful scenarios and inform mechanistically driven interventions designed to reduce the magnitude of the problem, as well as practice decisions related to case prioritization, triaging, and risk assessment.

Innovation and Translational Significance: This study advances knowledge about the conditions that increase the severity of elder abuse. A severity lens addresses limitations of conventional binary elder abuse conceptualizations by accounting for the complexity of cases and the range of lived experiences among survivors. This study found that various physical, psychological, and socio-cultural factors influence severity. Most consistently across subtypes, older adults who live with their perpetrator or have experienced child maltreatment are at heightened risk of experiencing severe elder abuse. Findings from this study will help identify older adults in particularly dangerous scenarios and inform intervention approaches and practice decisions to address this problem.

## Background and objectives

Elder abuse (EA) is a serious and pervasive form of inter-personal/family violence across the life course. EA refers to an intentional act or failure to act by a person in a relationship of trust that causes or creates a risk of harm to an older adult (Hall et al., 2016). EA victimization is associated with serious consequences, including premature mortality, poor physical/mental health, and increased rates of healthcare ­utilization (Yunus et al., 2019). Approximately 14% of community-dwelling older adults, globally, and 10% in Canada and the U.S. experience some form of EA each year (Pillemer et al., 2016).

Over the past several years, scholars have called for an examination of EA through a lens of severity (Burnes et al., 2018; Lachs et al., 2021). A severity lens allows us to understand EA along a continuum that more realistically reflects the heterogeneity and complexity of EA across cases, as well as the range of lived experiences among survivors. This lens is consistent with recent shifts toward client-centered EA intervention approaches that emphasize older adult self-determination and support a range of case outcomes (Burnes et al., 2023; Makaroun et al., 2025).

Increased EA severity is associated with heightened risk of adverse health and psychosocial consequences among victims (Fisher & Regan, 2006). Therefore, understanding factors that elevate EA severity will enhance our capacity to identify EA victims in particularly dangerous scenarios. It will inform the development of targeted interventions designed to reduce the magnitude of the problem, as well as practice decisions related to case prioritization, triaging, and risk assessment. A recent influx of studies focusing on EA severity has occurred, including integration of severity into population-based study design (Wong et al., 2021) and EA measurement (Liu et al., 2022; Yi et al., 2019).

To capture the complex etiology of EA, risk factors are often examined from an ecological perspective (National Center for Injury Prevention and Control, 2002; National Research Council [NRC]). Accordingly, the Contextual Theory of Elder Abuse conceptualizes EA as grounded in individual (including historical experience), relational, community, and societal processes (Roberto & Teaster, 2017). For individual older adults, EA risk is conceptualized as resulting from physical, functional, cognitive, and psycho-emotional vulnerabilities contributing to status inequality and power imbalance in relation to a harmer (NRC, 2003). Recent studies have found that functional impairment and poor physical or psycho-emotional health among victims are associated with increased EA severity (Burnes et al., 2017; Donder et al., 2016; Vilar-Compte & Gaitán-Rossi, 2018). Broader EA literature also suggests that historical older adult factors, including their previous experience of trauma or child maltreatment, contribute to EA risk (Burnes et al., 2022).

Within the relational context, social support is conceptualized as protective (Roberto & Teaster, 2017), and recent research has found that lower satisfaction with social networks and higher levels of loneliness are associated with heightened EA severity (Donder et al., 2016). Although findings across individual studies are mixed, shared living reflects a relational dynamic conceptualized as increasing EA risk, as it provides greater, unhindered access to the victim (NRC, 2003). Victim-harmer cohabitation has been associated with greater EA severity (Burnes et al., 2017; Melchiorre et al., 2021). At the societal level, this study includes broader, pervasive socio-structural processes related to gender, age, race/ethnicity, socioeconomic status, and geo-context that may elevate EA severity risk by contributing to unequal power dynamics and social arrangements, conditions of everyday contextual stress, and/or lower access to resources.

Despite the recent increase in EA severity research, our understanding of severity risk factors remains limited. Studies have used clinical convenience samples carrying systematic bias and/or cross-sectional designs, limiting causal inferences. Addressing these methodological limitations, the current article advances the state of science surrounding EA severity risk factors using data from a longitudinal, population-based design capturing data across several ecological domains. We hypothesized that EA victims reporting a decline (change) over time in functional, physical, cognitive, psycho-emotional status, or social support; shared living with their harmer; history of child maltreatment; or marginalized socio-structural status will be at higher risk of increased EA severity (Burnes et al., 2017).

## Research design and methods

### Sample

The Canadian Longitudinal Study on Aging (CLSA) is one of the largest prospective, population-based cohort studies on aging worldwide. It uses telephone and in-person interviews with a national, stratified, random sample of over 50,000 adults aged 45 to 85 at baseline (Raina et al., 2009). The current study used data from the CLSA baseline (2011–2015) and follow-up 1 (2015–2018) data collection waves. The EA module (covering emotional, physical, and financial abuse) was integrated into the CLSA at follow-up 1 (not subsequent waves) and administered to older adults aged 65 or older. Thus, our analytic sample involved CLSA participants who completed both baseline and follow-up interviews and were identified as an EA victim in the past year using the EA module (*n *= 2,364) (Burnes et al., 2022) as described in detail below. The CLSA yielded an overall participation rate of 50% (Raina et al., 2018). To mitigate concerns of generalizability, data were weighted. Weighted CLSA data are generalizable to the Canadian population on key variables such as age and sex; however, the CLSA sample is more educated, has slightly higher income levels, and has a higher proportion of older adults identifying as White (Raina et al., 2019). Baseline exclusion criteria were older adults living in institutions, three Canadian territories, or First Nations reserves/settlements; members of the Armed Forces; temporary VISA holders or transitional health coverage; cognitive impairment; or unable to respond in English/French. Individuals who became institutionalized between baseline and follow-up were included in follow-up interviews through personal or proxy-based interviewing (Raina et al., 2019).

### Dependent variables

EA was defined as events perpetrated by a person in a relationship involving an expectation of trust, including a family member, friend, caregiver, or other person considered close/trusting. Using recognized procedures to maximize sensitivity in epidemiological interpersonal/family violence research (NRC, 2003), each EA subtype was assessed using multiple, behaviorally defined items describing specific mistreatment behaviors. The EA module used a modified version of the Conflict Tactics Scale (CTS) to assess elder emotional (criticism, insulting, exclusion/ignoring, threats/intimidation) and physical (pushing/shoving/grabbing, throwing, hitting/slapping, trying to hit with something, choking, threatening to use a weapon) abuse. Financial abuse was measured using a validated, three-item tool (McDonald & Thomas, 2013), including items related to someone: making the older adult give money/possessions/property, taking money/possessions/property, and deliberately preventing access to money/possessions/property against the older adult’s will.

An affirmative response to any EA mistreatment behavior item initiated a follow-up question about past-year frequency (once, a few times, many times, every day/almost every day). Physical and financial abuse victimization were deemed present if an older adult affirmed one or more mistreatment subtype behaviors in the past year, regardless of frequency. To enhance emotional abuse measurement specificity (Pillemer et al., 2016), this subtype was deemed present if the criticism, insulting, or exclusion/ignoring items were affirmed with past-year frequency of at least ‘many times,’ or the threats/intimidation item was affirmed with any level of frequency (Burnes et al., 2022).

Calculation of mistreatment severity followed scoring systems used for the CTS (Strauss, 1995), Assessment of Self-Neglect Severity Scale (Dong et al., 2012), and prior EA severity research (Burnes et al., 2017). Combining severity dimensions of *multiplicity* and *frequency*, severity was measured continuously for each subtype by summing both the number of mistreatment behaviors affirmed and their past-year frequency (once = 1, a few times = 2, many times = 3, and every/almost every day = 4).

### Independent variables

#### Individual context

*Functional impairment* was measured continuously (range: 0–13) as the number of activities of daily living (ADL) or instrumental ADL (IADL) an older adult required help with, using a modified version of the Older Americans’ Resources and Services (OARS) Multidimensional Functional Assessment Questionnaire (Fillenbaum & Smyer, 1981). *General perceived health* measured an older adult’s reported health rating as poor (poor, fair) or good (good, very good, excellent). *Health conditions* were measured continuously (0-38) as the number of long-term/chronic health conditions diagnosed by a doctor (see Supplementary Text in Supplementary Material for the list of conditions). *Cognitive vulnerability* was assessed continuously using the Rey Auditory Verbal Learning Test (range: 0–15). For each of these variables, a dichotomous change variable was created characterizing whether an older adult’s score or status from baseline to follow-up remained the same/improved or declined.

*Depressive symptoms* were measured continuously (range: 0–30) using the Center for Epidemiologic Studies Short Depression Scale (CESD-10). A change variable assessed whether depression status (based on CESD-10 cutoff scores) remained the same/improved or declined (depression worsened) from baseline to follow-up. *Satisfaction with life* was measured continuously (range: 5–35) using the Satisfaction with Life Scale (SWLS) (Diener et al., 1985); a change score assessed whether status as satisfied/dissatisfied (based on SWLS cutoff scores) remained the same/improved or declined from baseline to follow-up. *Posttraumatic Stress Disorder* (PTSD) was measured continuously (range: 0–4) at baseline using the Primary Care Posttraumatic Stress Disorder screening instrument. PTSD was not assessed at follow-up; therefore, a change variable could not be generated.

*Childhood maltreatment* was measured using a modified version of the Childhood Experiences of Violence Questionnaire (Walsh et al., 2008), assessing nine maltreatment behaviors before age 16. It was measured continuously (range: 9–45), summing the number of different behaviors experienced and their frequency (never = 0, 1/2 times = 1, 3 to 5 times = 4, 6 to 10 times = 8, more than 10 times = 15).

#### Relational

*The number of cohabitants* was measured continuously as the number of cohabitants living in the household; a change variable measured whether the number of cohabitants from baseline to follow-up remained the same/decreased or increased. *Marital status* was assessed using categories of married/partnered, single/never married, widowed, or divorced/separated; a change variable indicated whether an older adult became widowed or not (other). *Perpetrator cohabitation* assessed whether the older adult victim lived with their harmer at the time of reported EA. It was assessed by asking the older adult directly if the person identified as perpetrating the mistreatment lived with them at the time of the mistreatment.

Total *social support* was measured continuously (range: 0–100) using the Medical Outcomes Study Social Support Survey. A change variable assessed whether high or low social support status, determined using the validated “optimal cut point” technique (Thiele & Hirschfeld, 2021), remained the same/improved or declined from baseline to follow-up. *Social contact with support network* assessed the frequency of contact with an older adult’s support network outside the home; a change variable assessed whether status as low (less than once a year/yearly/monthly) or high (weekly/daily) remained the same/improved or declined from baseline to follow-up. *Using internet to access websites* measured the frequency of internet access; a change variable assessed whether the status as low (never/yearly/monthly) or high (weekly/daily) remained the same/improved or declined from baseline to follow-up.

#### Societal

*Socio-structural* variables included sex (male, female), age (65–74, 75–84, 85+), race/culture (White, Asian, Black, other), and geo-cultural context (urban, rural). Income need measured the extent that current income was adequate in satisfying basic needs; a change variable assessed whether status as adequate (very well/adequate) or inadequate (some difficulty/not well/totally inadequate) remained the same/improved or declined from baseline to follow-up. As a note, our study did not capture factors situated in the *Community* level of the Contextual Theory of Elder Abuse.

### Analysis

Wherever possible, our analysis used independent change variables to confirm the direction of change underlying potential risk factors preceding victimization reports. Bivariate, unadjusted multinomial logistic regression was conducted on independent variables individually to explore relationships with EA severity. Multivariable regression models were then applied to independent variables simultaneously; selection of independent variables into multivariable models was based on significance in the unadjusted analyses (*p* < .10) (see Supplementary Table 1 in Supplementary Material for unadjusted analyses). However, all multivariable models controlled for sex, race/culture, and age, and whether the interview was conducted in person or via telephone. Severity outcome distributions violated parametric assumptions for ordinary least squares regression. Therefore, continuous severity scores were converted into ordinal outcomes (mild, moderate, high) by dividing the cumulative distribution of outcome scores into approximate thirds. The parallel lines test of proportional odds was violated with ordinal regression. Therefore, a less restrictive multinomial logistic regression approach was used. Analysis was undertaken for aggregate EA and for emotional abuse, physical abuse, and financial abuse subtypes separately.

## Results

### Sample characteristics

Table 1 presents descriptive characteristics of the older adult victim sample. Women (50.3%) and men (49.7%) were similarly represented. The majority of victims identified as White (95.9%), between the ages of 65 and 74 (63.9%), living in urban settings (87.9%), and having income that satisfied basic needs (87.7%). Among the total sample, emotional abuse was most common (88.1%), followed by financial (13.4%) and physical (12.6%).

**Table 1.** Descriptive characteristics of analytic elder abuse victim sample.

Characteristic	Item	Sample (*n *= 2,364)
**Individual**		
*Physical*	Functional impairment (0–13)	0.52 (1.21)
	General perceived health change	
	Poor	443 (18.7)
	Good	1,908 (80.7)
	Health conditions (0–38)	6.24 (3.58)
*Cognitive*	Rey auditory verbal learning test (0–15)	6.24 (3.58)
*Psycho-emotional*	Depressive symptoms (0–30)	7.45 (5.57)
	Posttraumatic stress disorder (0–4)	0.59 (1.04)
	Satisfaction with life (5–35)	25.34 (7.1)
*Childhood adversity*	Child maltreatment score (range: 0–135)	16.78 (24.03)
Relational		
*Home*	Number of cohabitants change (0–7)	0.88 (0.81)
	Marital status	
	Married/partnered	1,516 (64.1)
	Single/never married	168 (7.1)
	Widowed	304 (12.9)
	Divorced/separated	369 (15.8)
	Perpetrator cohabitation	
	No	1,075 (45.5)
	Yes	1,148 (48.6)
*Social*	Social support (0–100)	75.56 (19.9)
	Social contact with support network	
	Less than once/year, yearly, or monthly	113 (4.8)
	Weekly or daily	2,250 (95.2)
	Using internet to access websites	
	Never, yearly, or monthly	546 (23.1)
	Weekly or daily	1,806 (76.4)
**Society**		
*Socio-structural*	Sex	
	Male	1,174 (49.7)
	Female	1,190 (50.3)
	Age	
	65–74	1,510 (63.9)
	75–84	735 (31.1)
	85+	119 (5.0)
	Race/ethnicity	
	White	22 (96.1)
	Black	10 (0.4)
	Asian	29 (1.2)
	Other	50 (2.1)
	Income need satisfaction	
	Adequately satisfied basic needs	2,046 (87.7)
	Inadequately satisfies basic needs	286 (12.3)
	Geo-cultural context	
	Urban	2,079 (87.9)
	Rural	285 (12.1)
**Control**	Interview mode	
	Telephone	797 (33.7)
	In person	1,567 (66.3)
**Elder mistreatment**	Subtype	
	Emotional	2,083 (88.1)
	Physical	297 (12.6)
	Financial	320 (13.4)

*Note.* Mean (*SD*) reported for continuous variables, and *n* (%) reported for categorical variables.

### Risk factors

Tables 2 and 3 present results from the multinomial logistic regression models examining EA severity risk factors. The most consistent risk factor for EA severity across subtypes was perpetrator cohabitation. Living with the perpetrator was associated with significantly greater odds of highly severe aggregate (odds ratio [OR]: 2.00, 95% confidence interval [CI]: 1.58–2.53), emotional (OR: 1.76, CI: 1.32–2.34), physical (OR: 2.95, CI: 1.38–6.27), and financial (OR: 49.24, CI: 11.71–157.38) abuse. Child maltreatment was also a consistent predictor; each unit increase of child maltreatment (scale: 9–35) was associated with highly severe aggregate (OR: 1.02, CI: 1.01–1.02), emotional (OR: 1.02, CI: 1.01–1.02), and physical (OR: 1.02, CI: 1.01–1.04) abuse.

**Table 2.** Bivariate and multivariate multinomial regression models of change variables (from baseline to follow-up) predicting past-year aggregate elder abuse severity at follow-up.

Characteristic	Elder abuse aggregate (*n *= 1,840)			
	*Bivariate models*		*Multivariate model*	
	Moderate	High	Moderate	High
	OR (95%CI)	OR (95%CI)	OR (95%CI)	OR (95%CI)
Individual				
*Physical*				
Functional impairment change (ref: same/improved)				
Decline	0.96 (0.72–1.28)	1.13 (0.87–1.47)	–	–
General perceived health change (ref: same/improved)				
Decline	1.32 (0.94–1.86)	1.32 (0.96–1.83)	1.07 (0.71–1.63)	1.12 (0.75–1.68)
Health conditions change (ref: same/improved)				
Decline	1.10 (0.88–1.39)	1.33 (1.08–1.65)**	1.06 (0.81–1.38)	1.03 (0.80–1.33)
Missing^a^	1.74 (1.28–2.36)***	1.70 (1.27–2.28)***	1.41 (0.96–2.05)	1.45 (1.0–2.08)
*Cognitive*				
Rey auditory verbal learning test change (ref: same/improved)				
Decline	1.14 (0.90–1.45)	1.02 (0.81–1.28)	–	–
Missing^a^	0.80 (0.59–1.08)	1.16 (0.89–1.51)	–	–
*Psycho-emotional*				
Depressive symptoms change (ref: same/improved)				
Decline	1.55 (1.11–2.15)**	1.57 (1.15–2.13)**	1.42 (0.96–2.06)	1.46 (1.01–2.11)*
Posttraumatic stress disorder (range: 0–4, higher scores indicate higher indication of PTSD)	1.20 (1.08–1.34)***	1.47 (1.33–1.62)***	1.15 (1.01–1.31)*	1.29 (1.15–1.45)***
Satisfaction with life change (ref: same/improved)				
Decline	1.06 (0.67–1.67)	2.11 (1.44–3.08)***	0.94 (0.56–1.59)	1.53 (0.96–2.45)
*Childhood adversity*				
Child maltreatment score (range: 0–135, higher scores indicate more experience of child maltreatment)	1.01 (1.01–1.02)***	1.02 (1.01–1.02)***	1.01 (1.01–1.02)***	1.02 (1.01–1.02)***
Relational				
*Home*				
Number of co-habitants change (ref: same/improved)			–	–
Increased	1.08 (0.72–1.62)	0.86 (0.58–1.28)		
Marital status (ref: other)			–	–
Became widowed	1.39 (0.67–2.89)	1.53 (0.78–3.00)		
Perpetrator co-habitation (ref: no)				
Yes	1.99 (1.60–2.48)***	1.82 (1.49–2.23)***	2.01 (1.58–2.57)***	2.00 (1.58–2.53)***
*Social*				
Social support change (ref: same/improved)				
Decline	1.53 (1.11–2.10)**	1.47 (1.09–2.00)*	1.42 (0.98–2.05)	1.30 (0.91–1.88)
Missing^a^	0.92 (0.99–1.01)	0.98 (0.69–1.38)	0.94 (0.56–1.59)	1.02 (0.63–1.65)
Social contact with support network change (ref: same/improved)				
Decline	1.67 (0.96–2.92)	2.38 (1.44–3.92)***	1.59 (0.83–3.05)	1.92 (1.05–3.52)*
Using internet to access websites change (ref: same/improved)				
Decline	1.61 (1.01–2.55)*	1.55 (1.00–2.41)*	2.75 (1.52–4.97)***	2.21 (1.23–3.98)**
Societal				
*Socio-structural*				
Sex (ref: male)				
Female	1.05 (0.85–1.29)***	1.97 (1.62–2.93)***	0.97 (0.76–1.24)	1.89 (1.49–2.39)***
Age (ref: 65–74)				
75–84	0.86 (0.68–1.08)	0.61 (0.48–0.76)***	0.94 (0.72–1.23)	0.58 (0.44–0.76)***
85+	0.82 (0.40–1.68)	0.64 (0.32–1.27)	0.95 (0.72–1.24)	0.79 (0.43–1.46)
Race/culture (ref: White)				
Asian	1.06 (0.34–3.26)	0.70 (0.22–2.24)	0.68 (0.17–2.70)	0.90 (0.23–3.54)
Black	6.39 (1.04–39.18)*	1.05 (0.11–10.24)	4.21 (0.62–28.47)	0.50 (0.05–5.56)
Other	1.57 (0.71–3.47)	1.45 (0.68–3.09)	0.92 (0.36–2.35)	1.17 (0.50–2.75)
Income needs change (ref: same/improved)				
Decline	0.88 (0.55–1.39)	1.22 (0.82–1.81)	–	–
Geo-cultural context (ref: rural)				
Urban	0.86 (0.63–1.17)	0.94 (0.70–1.26)	–	–
Control				
Interviewed mode (ref: telephone)				
In-person	1.13 (0.91–1.42)	0.85 (0.70–1.05)	1.13 (0.86–1.47)	0.86 (0.67–1.10)

*Note.* CI = confidence interval. The multinomial referent category was *Mild*. The multinomial regression model satisfied the Likelihood Ratio model fit test (*p* < .001). A missing category was created for variables with 10% or more missing values to avoid exclusion of excess cases from models.

* *p *≤ .05.

** *p *< .01.

*** *p *< .001.

**Table 3.** Multivariate regression models of change variables (from baseline to follow-up) predicting past-year elder abuse subtype (emotional, physical, and financial) severity at follow-up.

Characteristic	Emotional abuse (*n *= 1,640)		Physical abuse (*n *= 221)		Financial abuse (*n *= 234)	
	Moderate OR (95%CI)	High OR (95%CI)	Moderate OR (95%CI)	High OR (95%CI)	Moderate OR (95%CI)	High OR (95%CI)
Individual						
*Physical*						
Functional impairment change (ref: same/improved)						
Decline	–	–	3.74 (1.48–9.44)**	1.38 (0.46–4.16)	–	–
General perceived health change (ref: same/improved)						
Decline	1.18 (0.80–1.76)	0.86 (0.53–1.40)	–	–	1.67 (0.51–5.44)	5.84 (1.34–25.47)*
Health conditions change (ref: same/improved)						
Decline	0.95 (0.74–1.23)	1.06 (0.77–1.45)	1.50 (0.65–3.47)	0.55 (0.23–1.30)	1.08 (0.44–2.65)	0.40 (0.12–1.30)
Missing^a^	1.28 (0.88–1.88)	1.75 (1.12–2.72)*	0.46 (0.14–1.47)	0.59 (0.21–1.65)	0.90 (0.33–2.51)	0.50 (0.14–1.78)
*Cognitive*						
Rey auditory verbal learning test 1 change (ref: same/improved)						
Decline	–	–	–	–	4.22 (1.46–12.19)**	2.76 (0.87–8.73)
Missing^a^	–	–	–	–	2.30 (0.96–5.50)	15.26 (4.09–57.00)***
*Psycho-emotional*						
Depressive symptoms change (ref: same/improved)						
Decline	1.47 (1.0–2.16)*	2.00 (1.30–3.07)**	–	–	2.50 (0.92–6.77)	1.08 (0.28–4.19)
Posttraumatic stress disorder (range: 0–4, higher scores indicate higher indication of PTSD)	1.24 (1.10–1.41)***	1.37 (1.19–1.57)***	0.76 (0.52–1.10)	1.05 (0.76–1.45)	0.96 (0.70–1.32)	1.13 (0.77–1.65)
Satisfaction with life change (ref: same/improved)						
Decline	1.10 (0.68–1.78)	1.57 (0.92–2.67)	–	–	8.53 (1.0–75.51)*	9.24 (1.06–80.29)*
*Childhood adversity*						
Child maltreatment score (range: 0–135, higher scores indicate more experience of child maltreatment)	1.01 (1.00–1.01)**	1.02 (1.01–1.02)***	1.01 (1.0–1.03)	1.02 (1.01–1.04)**	1.01 (0.99–1.02)	1.01 (0.99–1.03)
Relational						
*Home*						
Number of co-habitants change (ref: same/improved)						
Increased	–	–	–	–	–	–
Marital status (ref: other)						
Became widowed	–	–	–	–	–	–
Perpetrator co-habitation (ref: no)						
Yes	1.65 (1.30–2.09)***	1.76 (1.32–2.34)***	1.65 (0.78–3.48)	2.95 (1.38–6.27)**	7.87 (2.44–25.37)***	42.94 (11.71–157.38)***
*Social*						
Social support change (low/high) (ref: same/improved)						
Decline	1.16 (0.80–1.67)	1.34 (0.88–2.06)	2.08 (0.69–6.32)	4.21 (1.39–12.78)**	–	–
Missing^a^	1.34 (0.82–2.20)	1.19 (0.66–2.16)	5.23 (0.99–27.66)*	2.13 (0.30–14.86)	–	–
Social contact with support network change (ref: same/improved)						
Decline	0.89 (0.50–1.60)	1.22 (0.65–2.29)	–	–	0.49 (0.03–8.56)	9.83 (1.26–76.79)*
Using internet to access websites change (ref: same/improved)						
Decline	2.75 (1.52–4.97)***	1.57 (0.77–3.19)	0.73 (0.13–4.25)	4.57 (1.21–17.20)*	0.13 (0.01–1.38)	1.98 (0.46–8.62)
Societal						
*Socio-structural*						
Sex (ref: male)						
Female	1.35 (1.07–1.72)*	2.27 (1.70–3.05)***	0.50 (0.24–1.06)	0.77 (0.36–1.64)	1.25 (0.58–2.68)	0.82 (0.30–2.23)
Age (ref: 65–74)						
75–84	0.89 (0.68–1.16)	0.65 (0.46–0.92)*	–	–	0.78 (0.36–1.73)	0.55 (0.18–1.69)
85+	0.75 (0.37–1.54)	1.22 (0.55–2.66)	0.92 (0.40–2.09)	0.51 (0.21–1.28)	0.90 (0.20–4.0)	1.15 (0.12–10.70)
Race/culture (ref: White)						
Asian	0.93 (0.29–2.97)	0.20 (0.01–4.68)	–	–	–	–
Black	1.25 (0.09–18.40)	1.93 (0.09–42.70)	–	–	–	–
Other	0.78 (0.31–1.96)	1.80 (0.71–4.59)	0.09 (0–2.17)	0.75 (0.14–4.12)	0.12 (0.02–0.88)*	0.12 (0.02–0.78)*
Income needs change (ref: same/improved)						
Decline	–	–	–	–	0.26 (0.03–2.75)	6.14 (1.05–36.14)*
Geo-cultural context (ref: rural)						
Urban	–	–	–	–	–	–
Control						
Interviewed mode (ref: telephone)						
In-person	1.02 (0.79–1.32)	0.74 (0.54–0.99)*	1.25 (0.54–2.89)	0.66 (0.29–1.49)	1.30 (0.59–2.84)	0.46 (0.18–1.21)

*Note.* CI = confidence interval. The multinomial referent category was *Mild*. The multinomial regression model satisfied the Likelihood Ratio model fit test (*p* < .001). The Race/Culture (White, non-White) and Age (65–74, 75+) variables were collapsed into two categories in the physical abuse model due to sparseness. A missing category was created for variables with 10% or more missing values to avoid exclusion of excess cases from models. All models satisfied the Likelihood Ratio goodness of fit test (*p *< .05).

* *p *≤ .05.

** *p *< .01.

*** *p *< .001.

From the physical domain, declining functional capacity was significantly associated with moderately severe physical abuse (OR: 3.74, CI: 1.48–9.44), and declined self-reported health significantly predicted severe financial abuse (OR: 5.84, CI: 1.34–25.47).

Psycho-emotionally, a decline in depression and PTSD status were significantly associated with highly severe aggregate (OR: 1.46, CI: 1.01–2.11; OR: 1.29, CI: 1.15–1.45) and emotional (OR: 2.00, CI: 1.30–3.07; OR: 1.37, CI: 1.19–1.57) abuse, while declining life satisfaction was associated with highly severe financial abuse (OR: 9.24, CI: 1.06–80.29).

Socially, declined internet usage was significantly associated with highly severe aggregate (OR: 2.21, CI: 1.23–3.98) and physical (OR: 4.57, CI: 1.21–17.20) abuse, as well as moderate emotional abuse (OR: 2.75, CI: 1.52–4.97). Decline in social network contact was significantly associated with highly severe aggregate (OR: 1.92, CI: 1.05–3.52) and financial (OR: 9.83, CI: 1.26–76.79) abuse, while declined total social support was associated with highly severe physical abuse (OR: 4.21, CI: 1.39–12.78).

Socio-demographically, women were significantly more likely to experience highly severe aggregate (OR: 1.89, CI: 1.49–2.39) and emotional (OR: 2.27, CI: 1.70–3.05) abuse. Older adults reporting a decline in income as meeting basic needs were significantly more likely to experience highly severe financial abuse (OR: 6.14, CI: 1.05–36.14).

## Discussion and implications

This study sought to advance knowledge about risk factors for EA severity using data from a longitudinal, population-based study. Compared with the conventional binary (yes/no) conceptualization of EA, severity measures more accurately capture the spectrum of experiences among victims and reflect the complex reality of EA intervention work. Findings from this study identified EA severity risk factors across individual (physical, psycho-emotional, historical), relational (home, social), and societal (socio-structural) contexts and found varying risk profiles across each mistreatment type.

The most consistent risk factor for EA severity across subtypes was perpetrator cohabitation (relational context), consistent with prior research (Burnes et al., 2017; Melchiorre et al., 2021). A shared victim-perpetrator living arrangement may provide greater, unmonitored opportunities for mistreatment and close-quarter conditions to facilitate escalation in frequency/intensity. Shared living also allows a perpetrator to socially isolate the victim through gatekeeping tactics and by controlling contact with people outside the home. Although victim-perpetrator separation may seem a logical choice in some cases, older adults often do not wish to move living settings themselves or evict their harmer (Burnes et al., 2024), who may be dependent on them or vice versa (Roberto et al., 2025). In such cases, intervention strategies need to include appropriate safety planning and measures that mitigate risk attached to shared living, such as reducing co-dependence, introducing frequent visitors into the home, and/or increasing older adult participation in activities outside of the home. Indeed, different forms of social support/contact were found as important determinants of EA severity across each mistreatment subtype in this study. Internet usage was also found to be important in mitigating EA severity risk, and online forms of connection to informal/formal supports may serve a protective function.

At the individual level, prior experience of child maltreatment was consistently associated with EA severity in later life. This finding extends a growing body of research that links child maltreatment to EA in the general population (Burnes et al., 2022), as well as research that links family violence across several developmental periods throughout the life course (Herrenkohl et al., 2022). The mechanisms that link family violence across developmental periods of life are not yet established. However, several theories focusing on psychobiological, attachment, social learning, and social information processes provide promising pathways, including those related to stress response, internalized relational working models, conflict-oriented patterns of cognition and learned behavior, as well as processes of violence acceptance and normalization (Herrenkohl et al., 2022). Further research is required to understand how and why interpersonal violence is connected across the life course.

The current study contained limitations. Without EA data at both baseline and follow-up, it is not possible to identify risk factors for increasing severity over time. Our operationalization of severity is limited to dimensions of frequency and multiplicity; future research should develop a comprehensive severity measure that accounts for other potentially important dimensions (e.g., intensity, harm, subjective victim appraisal). Similarly, future studies could include more nuanced measures of change in physical and financial health status. The CLSA sample was biased toward older adults with higher levels of education and income and identify as White, and, in turn, does not fully reflect the diversity of the Canadian population.

Overall, however, the current study represents the most methodologically rigorous examination of EA severity risk to date. Using a longitudinal, population-based design, this study found EA severity risk factors that span individual, historical, relational, and societal levels of ecological influence. Findings will enhance our capacity to identify EA victims in particularly dangerous circumstances. They will also inform the development of interventions designed to prevent EA and reduce harm. This study contributes to a growing body of EA scholarship that aims to advance our understanding of EA phenomena beyond conventional binary terms.

## Supplementary material

Supplementary data are available at *Innovation in Aging* online.

## Supplementary Material

igaf101_Supplementary_Data

## Data Availability

The data underlying this article are available in the Canadian Longitudinal Study on Aging at www.clsa-elcv.ca for researchers who meet the criteria for access to de-identified CLSA data. This study was not preregistered.
