# Supplementary material for: Risk factors for elder abuse severity: findings from the Canadian longitudinal study on aging
Source: Innov Aging. 2025 Sep 23;9(10):igaf101. doi: 10.1093/geroni/igaf101 (PMC12596471; doi:10.1093/geroni/igaf101)
Supplement: igaf101_Supplementary_Data [file igaf101_supplementary_data.zip › innage suppl Burnes, Rotsaert, Lachs, & Pillemer.docx]

***Innovation in Aging* Supplementary Material: Burnes, Rotsaert, Lachs, & Pillemer. Risk Factors for elderabuse severity: Findings from the Canadian Longitudinal Study on Aging.**

**Supplementary Text: List of Chronic Health Conditions**

Health conditions included in the survey included: Osteoarthritis–knee, osteoarthritis–hip, osteoarthritis–hands, rheumatoid arthritis, arthritis–other, asthma, lung condition, high blood pressure/hypertension, diabetes, heart disease, angina, heart attack, peripheral vascular disease, stroke/CVA, transient ischemic attack, memory problem, Alzheimer’s disease, Parkinson’s disease, multiple sclerosis, epilepsy, migraine headaches, stomach ulcers, bowel disorder, bowel incontinence, urinary incontinence, cataracts, glaucoma, macular degeneration, cancer, mood disorder, anxiety disorder, allergies, osteoporosis, back problems, under-active thyroid gland, over-active thyroid gland, kidney disease, and other long-term conditions.

**Supplementary Table 1.** Bivariate regression models of change variables (from baseline to follow-up) predicting past-year elder abuse subtype (emotional, physical, financial) severity at follow-up

| **Characteristic** | **Emotional Abuse** | | **Physical Abuse** | | **Financial Abuse** | |
| --- | --- | --- | --- | --- | --- | --- |
|  | ***Bivariate Multinomial Models***  (n = 2083) | | ***Bivariate Multinomial Models***  (n = 297) | | ***Bivariate Multinomial Models***  (n = 320) | |
|  | Moderate  OR (95%CI) | High  OR (95%CI) | Moderate  OR (95%CI) | High  OR (95%CI) | Moderate  OR (95%CI) | High  OR (95%CI) |
| **INDIVIDUAL** | | | | | |  |
| ***Physical*** | | | | | |  |
| Functional Impairment Change (Ref: Same/Improved) |  |  |  |  |  |  |
| Decline | 0.99 (0.74-1.30) | 1.30 (0.95-1.78) | 2.74 (1.39-5.40)** | 0.91 (0.38-2.14) | 1.40 (0.73-2.67) | 0.85 (0.40-1.78) |
| General Perceived Health Change (Ref: Same/Improved) |  |  |  |  |  |  |
| Decline | 1.56 (1.12-2.18)** | 1.15 (0.77-1.72) | 1.25 (0.60-2.61) | 0.91 (0.39-2.10) | 2.19 (0.93-5.19)+ | 1.87 (0.76-4.58) |
| Health Conditions Change (Ref: Same/Improved) |  |  |  |  |  |  |
| Decline | 1.01 (0.81-1.26) | 1.33 (1.02-1.72)* | 0.96 (0.51-1.79) | 0.50 (0.25-0.98)* | 1.92 (1.02-3.59)* | 1.52 (0.82-2.84) |
| Missing^a^ | 1.65 (1.21-1.25)** | 2.08 (1.46-2.98)*** | 0.73 (0.35-1.54) | 0.56 (0.26-1.21) | 0.98 (0.49-1.99) | 0.77 (0.38-1.56) |
| ***Cognitive*** | | | | | |  |
| Rey Auditory Verbal Learning Test 1 Change (Ref: Same/Improved) |  |  |  |  |  |  |
| Decline | 1.12 (0.89-1.42) | 1.20 (0.91-1.58) | 0.66 (0.35-1.22) | 0.81 (0.43-1.55) | 1.89 (1.0-3.57)* | 2.53 (1.33-4.82)** |
| Missing^a^ | 1.00 (0.75-1.33) | 1.16 (0.84-1.61) | 0.88 (0.42-1.84) | 0.61 (0.26-1.46) | 3.41 (1.62-7.19)*** | 4.66 (2.20-9.86)*** |
| ***Psycho-Emotional*** | | | | | |  |
| Depressive Symptoms Change (Ref: Same/Improved) (Ref: Same/Improved) |  |  |  |  |  |  |
| Decline | 1.59 (1.14-2.20)** | 1.90 (1.32-2.72)*** | 0.87 (0.36-2.07) | 0.71 (0.26-1.92) | 2.84 (1.34-6.01)** | 1.38 (0.59-3.21) |
| Posttraumatic Stress Disorder (range: 0-4, higher scores indicate higher indication of PTSD) | 1.26 (1.14-1.40)*** | 1.54 (1.38-1.73)*** | 0.67 (0.52-0.87)** | 1.06 (0.85-1.31) | 0.99 (0.79-1.24) | 1.22 (1.0-1.50)+ |
| Satisfaction with Life Change (Ref: Same/Improved) |  |  |  |  |  |  |
| Decline | 1.31 (0.87-1.98) | 2.30 (1.50-3.52)*** | 1.54 (0.50-4.74) | 2.44 (0.82-7.29) | 4.57 (1.04-20.12)* | 11.93 (3.03-47.07)*** |
| ***Childhood Adversity*** |  |  |  |  |  |  |
| Child Maltreatment Score (range: 0-135, higher scores indicate more experience of child maltreatment) | 1.01 (1.00-1.01)*** | 1.02 (1.01-1.02)*** | 1.0 (0.99-1.01) | 1.01 (1.0-1.03)* | 1.01 (1.0-1.02) | 1.01 (1.0-1.02)* |
| **RELATIONAL** | | | | | |  |
| ***Home*** | | | | | |  |
| Number of Co-Habitants Change (Ref: Same/Improved) |  |  |  |  |  |  |
| Increased | 0.99 (0.67-1.48) | 0.77 (0.47-1.26) | 0.42 (0.07-2.48) | 0.73 (0.15-3.69) | 0.72 (0.26-1.95) | 0.66 (0.23-1.85) |
| Marital Status (Ref: Other) |  |  |  |  |  |  |
| Became Widowed | 0.92 (0.44-1.93) | 1.41 (0.65-3.07) | 0.16 (0-7.55) | 0.20(0-9.98) | 0.77 (0.24-2.49) | 0.20 (0.03-1.43) |
| Perpetrator Co-Habitation (Ref: No) |  |  |  |  |  |  |
| Yes | 1.53 (1.24-1.88)*** | 1.53 (1.20-1.96)*** | 1.41 (0.77-2.59) | 2.64 (1.41-4.95)** | 2.08 (0.92-4.71)+ | 7.69 (3.66-16.14)*** |
| ***Social*** | | | | | |  |
| Social Support Change (Low/High) (Ref: Same/Improved) |  |  |  |  |  |  |
| Decline | 1.20 (0.88-1.64) | 1.54 (1.09-2.19)* | 2.68 (1.15-6.24)* | 2.48 (1.03-5.98)* | 1.29 (0.62-2.69) | 0.72 (0.32-1.67) |
| Missing^a^ | 1.37 (0.93-2.02) | 1.59 (1.03-2.46)* | 4.21 (1.50-11.79)** | 0.89 (0.20-3.90) | 0.71 (0.30-1.67) | 0.45 (0.17-1.19) |
| Social Contact with Support Network Change (Ref: Same/Improved) |  |  |  |  |  |  |
| Decline | 1.18 (0.71-1.94) | 1.68 (0.99-2.87)+ | 0.30 (0.04-2.26) | 2.31 (0.68-7.85) | 1.10 (0.19-6.28) | 6.38 (1.79-22.65)** |
| Using Internet to Access Websites Change (Ref: Same/Improved) |  |  |  |  |  |  |
| Decline | 1.73 (1.10-2.73)* | 1.47 (0.86-2.51) | 0.78 (0.18-4.42) | 4.70 (1.54-14.36)** | 0.25 (0.05-1.31) | 3.10 (1.37-7.03)** |
| **SOCIETAL** | | | | | |  |
| ***Socio-Structural*** | | | | | |  |
| Sex (Ref: Male) |  |  |  |  |  |  |
| Female | 1.43 (0.85-1.29)*** | 2.20 (1.62-2.93)*** | 0.47 (0.27-0.82)** | 0.83 (0.47-1.50) | 1.44 (0.84-2.47) | 2.31 (1.32-4.02)** |
|  |  |  |  |  |  |  |
| Age (Ref: 65-74) |  |  |  |  |  |  |
| 75-84 | 0.87 (0.70-1.10) | 0.66 (0.50-0.86)** | 1.86 (1.05-3.31)* | 0.68 (0.35-1.35) | 1.09 (0.62-1.94) | 0.35 (0.18-0.70)** |
| 85+ | 0.81 (0.38-1.73) | 0.77 (0.32-1.90) | 1.56 (0.29-8.47) | 0.29 (0.02-5.0) | 2.38 (0.62-9.19) | 0.70 (0.13-3.84) |
| Race/Culture (Ref: White) |  |  |  |  |  |  |
| Asian | 1.23 (0.44-3.43) | 0.18 (0.02-1.96) | - | - | - | - |
| Black | 0.95 (0.07-13.34) | 1.22 (0.07-22.01) | - | - | - | - |
| Other | 0.94 (0.43-2.08) | 1.67 (0.75-3.72) | 0.11 (0.01-2.05) | 1.71 (0.50-5.92) | 0.41 (0.11-1.56) | 0.32 (0.07-1.42) |
| Income Needs Change (Ref: Same/Improved) |  |  |  |  |  |  |
| Decline | 1.28 (0.83-1.98) | 1.09 (0.65-1.83) | 0.19 (0.03-1.06)+ | 0.44 (0.12-1.71) | 0.40 (0.07-2.26) | 4.11 (1.58-10.68)** |
| Geo-Cultural Context (Ref: Rural) |  |  |  |  |  |  |
| Urban | 1.00 (0.74-1.36) | 0.84 (0.60-1.18) | 1.38 (0.54-3.50) | 1.25 (0.47-3.37) | 1.06 (0.45-2.50) | 0.63 (0.29-1.36) |
| **CONTROL** |  |  |  |  |  |  |
| Interviewed Mode (Ref: Telephone) |  |  |  |  |  |  |
| In-Person | 1.07 (0.87-133) | 0.74 (0.58-0.94)* | 1.76 (0.94-3.31)+ | 0.79 (0.43-1.46) | 1.30 (0.74-2.29) | 0.54 (0.31-0.94)* |

Notes. CI = Confidence Interval; ** p* ≤ 0.05, ** *p* < 0.01, *** *p* < 0.001, † *p* < 0.10 (borderline). Multinomial referent category was *Mild*. The Race/Culture variable was collapsed into two categories in the physical and financial abuse models due to sparseness.
